# Identification of decompensation episodes in chronic heart failure patients based solely on heart sounds

**DOI:** 10.3389/fcvm.2022.1009821

**Published:** 2022-11-15

**Authors:** David Susič, Gregor Poglajen, Anton Gradišek

**Affiliations:** ^1^Department of Intelligent Systems, Jožef Stefan Institute, Ljubljana, Slovenia; ^2^Jožef Stefan Postgraduate School, Ljubljana, Slovenia; ^3^Advanced Heart Failure and Transplantation Program, Department of Cardiology, University Medical Centre Ljubljana, Ljubljana, Slovenia; ^4^Medical Faculty, University of Ljubljana, Ljubljana, Slovenia

**Keywords:** heart failiure, cardiac decompensation, heart sound, machine learing, phonocardiogram (PCG), artificial intelligence-AI, decompensation detection, classification

## Abstract

Decompensation episodes in chronic heart failure patients frequently result in unplanned outpatient or emergency room visits or even hospitalizations. Early detection of these episodes in their pre-symptomatic phase would likely enable the clinicians to manage this patient cohort with the appropriate modification of medical therapy which would in turn prevent the development of more severe heart failure decompensation thus avoiding the need for heart failure-related hospitalizations. Currently, heart failure worsening is recognized by the clinicians through characteristic changes of heart failure-related symptoms and signs, including the changes in heart sounds. The latter has proven to be largely unreliable as its interpretation is highly subjective and dependent on the clinicians’ skills and preferences. Previous studies have indicated that the algorithms of artificial intelligence are promising in distinguishing the heart sounds of heart failure patients from those of healthy individuals. In this manuscript, we focus on the analysis of heart sounds of chronic heart failure patients in their decompensated and recompensated phase. The data was recorded on 37 patients using two types of electronic stethoscopes. Using a combination of machine learning approaches, we obtained up to 72% classification accuracy between the two phases, which is better than the accuracy of the interpretation by cardiologists, which reached 50%. Our results demonstrate that machine learning algorithms are promising in improving early detection of heart failure decompensation episodes.

## Introduction

Chronic heart failure (CHF) is a complex chronic condition, characterized by the inability of the heart muscle to provide sufficient perfusion to meet the metabolic demands of the body; alternatively the failing heart stabilizes the circulation by operating at the higher filling pressures, which generate the majority of the symptoms and signs, characteristic of CHF. Globally, CHF has reached epidemic proportions, affecting roughly 2% of the world’s overall population, with the incidence increasing at 2% annually. The prevalence of CHF reaches around 10% in the overall population aged over 65 years and additionally carries a significant burden in terms of healthcare costs and personnel expenditure. Importantly, the prognosis of CHF patients remains dismal with 50% mortality at 5 years, which is largely related to heart failure decompensation episodes that require in-hospital management (1). Available literature suggests that identifying the CHF decompensation episodes in their pre-symptomatic phase (when the patient does not yet subjectively feel worse) may enable clinicians to make appropriate and timely changes to patient’s medical therapy thus preventing overt CHF decompensation to occur or to occur in much milder forms that do not require hospitalization (2). This may significantly improve patients’ outcomes (2). As microelectromechanical systems technology is invasive, of limited availability and, at least for now, prohibitively expensive, it has not yet exerted a wider impact on the management of heart failure. Importantly, studies using easily obtainable (but non-specific) clinical parameters (body weight, blood pressure, heart rate, etc.) displayed only limited success in accurately predicting CHF decompensation episodes. There is thus a significant unmet need in the heart failure community for effective, cost-efficient and robust protocols for early detection of CHF decompensation episodes. First automatic detections of CHF and other cardiovascular diseases were performed with electrocardiogram data (3, 4), heart rate variability data, photoplethysmogram, and clinical data such as respiratory rate, weight, pulse rate, age, and blood pressure (5). Recently, automated methods for analysing heart sounds and detecting cardiovascular disease from heart sounds have been increasingly developed as more and more datasets of heart sound recordings have become publicly available (6–8).

Heart sound classification algorithms found in the literature include classical ML models, statistical models, and artificial neural networks (NN) (9). There are a few papers that specifically address CHF. In the work of Gjoreski et al. (10), a stack of ML classifiers was used to classify normal sounds and heart failure. In the preliminary study, Gjoreski et al. (11) used a simple decision tree classifier to classify compensated and decompensated stages of CHF, using only the portion of our dataset recorded under the first experimental setup. Gao et al. (12) compared the fully convolutional NN, gated recurrent unit, long short-term memory, and support-vector machine (SVM) models to classify normal heart and two subtypes of CHF. Liu et al. (13) compared NN and SVM in classifying normal heart and subtype of heart failure. In the work of Zheng et al. (14), the SVM, NN, and a statistical hidden Markov model were compared in classifying normal and CHF sounds. In our first study, we tested ML algorithms for detecting decompensation in CHF using general audio features generated with a dedicated audio feature tool. Features were extracted from segments with a fixed length of 2 s from a subset of our current data set (15).

As previous studies (10, 11, 16) have demonstrated promising results in distinguishing between the heart sounds of healthy people from those of CHF patients, we now focus on a more specific task. In this manuscript, we explore how ML algorithms can be employed to identify decompensation episodes based on the heart sounds of CHF patients. The study was performed on the recordings of 37 patients in both decompensated and recompensated phases, using two types of electronic stethoscopes. We discuss the performances of several ML algorithms in view of feasibility of this approach for telemedicine application. We compare the classification accuracies of the ML models with that of cardiologists, who are domain experts.

## Materials and methods

### Data

Our dataset consists of phonocardiograms (PCG) of 37 CHF patients (average age of 51.3 ± 13.3 years). The dataset was obtained by two different setups. The first part (21 subjects) was obtained with a 3M*™* Littmann Electronic Stethoscope Model 3200 (17) digital stethoscope and consists of PCGs 30 s in length. The second part (16 subjects) was obtained with the Eko DUO ECG + Digital Stethoscope (18) and consists of PCGs 15 s in length. Both devices use built-in filters to reduce ambient noise and record single channel audio signals at a sampling rate of 4 kHz. According to the principal component analysis, the difference between the recordings from the two devices after preprocessing were small, thus it was reasonable to consider both as the same, device-independent dataset. The subjects were recorded in both the decompensated and the recompensated phase. The decompensated episode was recorded when the patient was admitted to the hospital for worsening heart failure episode, while the recompensated one was recorded upon discharge from the hospital when the patient was optimally recompensated and was deemed optivolemic. The PCGs were collected by medical professionals at University Medical Centre Ljubljana from left parasternal 3rd intercostal space body position. Overall, our dataset consists of 75 PCGs, 37 and 38 for compensated and decompensated phases, respectively, and adds up to 29 min and 15 s in length. The study protocol was reviewed and approved by the Republic of Slovenia National Medical Ethics Committee (decision number 0120-276/2016-5).

A phonocardiogram consists of regular S1 and S2 sounds, which are caused by the closing and opening of the heart valves, and several additional sounds that may be present. These include the S3 and S4 sounds, gallops, murmurs, opening snaps, rubs, and clicks. While the S3 sound may also be present in normal hearts of young children and athletes, other abnormal sounds are never present in a normal heart. The pathophysiology of the heart sounds can be found in Table 1.

**TABLE 1:** Pathophysiology of heart sounds (9).

Heart sound	Frequency range	Characteristics	Duration/Location
S1	10–200 Hz	Dull and prolonged	0.12–0.15 s
S2	20–250 Hz	Sharp and short	0.08–0.12 s
S3	25–70 Hz	Soft and thudding quality	0.04 s, early diastole
S4	15–70 Hz	Weak and rumbling	Slightly before S1
Gallop	15–50 Hz	Galloping rhythm	0.08–0.2 s, diastole
Murmurs	Up to 600 Hz	Whooshing, rumbling	Systole, diastole
Opening snaps	100–800 Hz	Snapping sound	Diastole
Rubs	100–800 Hz	Scratching sound	Systole, early/Late diastole
Clicks	100–800 Hz	Short and loud	Early systole

### Methods

The outcome of interest used to evaluate the ML models was a binary variable indicating whether a PCG represents a decompensated or a recompensated CHF phase. The steps of the methodology pipeline included preprocessing of the PCGs, feature extraction, and training and evaluation of the ML models.

#### Patient selection

We performed a prospective nonrandomized cohort study that included 37 consecutive patients hospitalized for worsening heart failure at the Advanced Heart Failure Center, Dept. of Cardiology, UMC Ljubljana. Inclusion criteria were as follows: chronic heart failure of ischemic or non-ischemic etiology, hospitalization for worsening heart failure <24 h, age >18 years; We did not consider patients with severe valvular disease, artificial valves, patients with acute myocardial infarction and/or de-novo acute heart failure, patients in cardiogenic shock, on vasoactive and/or inotropic support, on mechanical ventilation or on short- or long-term mechanical circulatory support for this analysis or patients that were hospitalized for worsening heart failure >24 h for this analysis. Clinical, biochemical and medical therapy data were collected for all the patients at the time of the initial heart sound sampling.

All patients included in this analysis were recompensated using levosimendan, followed by the intravenous diuretic therapy. In all study participants, heart sounds were recorded before the infusion of levosimendan (decompensated phase) and upon reaching the optivolemic phase (recompensated phase).

#### Preprocessing

The first part of the preprocessing step was filtering. Although heart sounds have frequencies of up to 800 Hz (see Table 1), the most dominant frequencies are in the frequency range of 20–400 Hz (19). The mean spectral roll-off frequency (frequency below which 85% of the total spectral energy lies) of our dataset is 49.9 ± 9.7 and 304.2 ± 99.4 Hz for the PCGs recorded by the first and the second experimental setting, respectively. To reduce the effects of different recording settings and to reduce noise, the PCGs were filtered with a bandpass Butterworth filter of order 4 and a frequency range from 25 to 400 Hz.

As the PCGs obtained by the two experimental settings were also recorded at different amplitudes, the next preprocessing step was heart sound signal normalization. We used the root mean square (RMS) normalization with the target amplitude of -20 dBFS. As opposed to the peak normalization, which normalizes the signal based on the highest peak, the RMS normalization normalizes the signal based on the average power level by calculating the average value of all peaks.

The Springer’s modification (20) of Schmidt’s method (21) was used to split the heart sound into separate cardiac cycles and to find the four main states of each segment (RR): S1, systole, S2, and diastole. This algorithm uses a hidden semi-Markov model and Viterbi decoding and provides a state-of-the-art method for segmenting heart sounds. Segmentation allows us to extract the features of the possible abnormal sounds from the corresponding heart sound states. In manually reviewing the segmented PCGs, we found that seven (9%) of the recordings either consisted of a significant number of segments that were not correctly determined, or the recording itself was so unclear that it was impossible to tell whether the segments were correct or not. The two most common reasons for the segmentation error were that one of the main sounds (S1 or S2) was not detected by the PCG recorder, resulting in a segment that was too long (longer than one RR interval), or that the high-amplitude noise was detected as one of the two main sounds, resulting in a segment that was too short (shorter than one RR interval).

**FIGURE 1:**
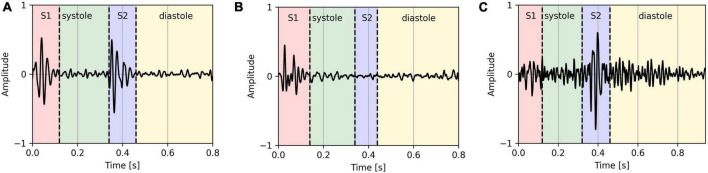
Clear PCG segment **(A)**, PCG segment with missing S2 sound **(B)**, and a noisy PCG segment **(C)**.

As some of the features are calculated based on the characteristics of the S1 and S2 sounds, the segments where either of the sounds was not present (based on the signal envelope) or the signal-to-noise ratio was too high were excluded in the analysis. On average, 3.3 2.6% segments per PCG recording were removed. Figure 1 shows an example of a clear segment, an example of a segment that was removed because S2 is missing, and an example of a segment that was removed because it is too noisy.

The normalization was performed using Python 3.7 (Python Programming Language, RRID:SCR_008394) and the library Pydub 0.25.1 (22), while the filtering and segmentation was performed using Matlab R2021a (MATLAB, RRID:SCR_001622) (23).

#### Feature extraction

A total of 177 features were extracted from each segment. These included features in the time domain, frequency domain, statistical features, and features generated by a 4-level wavelet decomposition. The complete list of features can be found in Table 2.

**TABLE 2:** List of features extracted from the phonocardiograms segments.

Feature type (N)	Per segment state	Domain	Description
BPM (1)	RR	Time	Inverse of segment duration in beats per minute
Dur_*state* (4)	S1, Sys, S2, Dia	Time	Duration in milliseconds
Dur_Ratio_*ratio* (8)		Time	Duration ratios
MeanEnv_Ratio_*ratio* (8)		Statistical	Mean envelope ratios
RMS_*state* (4)	S1, Sys, S2, Dia	Statistical	Root-mean-square of a signal
RMS_Ratio_*ratio* (8)		Statistical	Root-mean-square ratios
ZC_*state* (4)	S1, Sys, S2, Dia	Statistical	Zero crossings
SE_*state* (4)	S1, Sys, S2, Dia	Statistical	Sample entropy
Skewness_*state* (4)	S1, Sys, S2, Dia	Statistical	Skewness
Kurtosis_*state* (4)	S1, Sys, S2, Dia	Statistical	Kurtosis
PSD_*region*_*band* (24)	Sys, Dia	Frequency	Power spectral density for different frequency bands
mfcc1-13_*state* (52)	S1, Sys, S2, Dia	Frequency	13 Mel-frequency cepstral coefficients
SpecCentroid_*state* (4)	S1, Sys, S2, Dia	Frequency	Spectral centroid
SpecBandwidth_*state* (4)	S1, Sys, S2, Dia	Frequency	2nd order spectral bandwidth
SpecContrast2-5_*state* (16)	S1, Sys, S2, Dia	Frequency	Spectral contrast for different frequency bands
SpecFlatness_*state* (4)	S1, Sys, S2, Dia	Frequency	Spectral flatness
SpecRolloff_*state* (4)	S1, Sys, S2, Dia	Frequency	Frequency below which 85% of the total spectral energy lies
PolyFeatures_*state* (4)	S1, Sys, S2, Dia	Frequency	Coefficients of degree-1 polynomial fit to the spectrogram
dwt1-4_*state* (16)	S1, Sys, S2, Dia	Wavelet	Level 4 discrete wavelet transform coefficients

Features were extracted from the entire PCG signal data and from each of the four “*_states.”* In some cases, the features were calculated as a ratio of the features of the states. The “_*ratio”* features include S1/RR, Sys/RR, S2/RR, Dia/RR, S1/S2, Sys/Dia, Sys/S1, and Dia/S2. To extract the frequency domain features, the segments were transformed from the time domain to the frequency domain using a fast Fourier transform with a Hanning window of 64 milliseconds in length and a stride of 16 milliseconds. For the power spectral density frequency features, we selected frequency “*_bands”* of 25–40, 40–60, 60–80, 80–100, 100–120, 120–140, 140–160, 160–180, 180–200, 200–250, 250–300, and 300–400 Hz. The selected frequency bands are similar to those selected by the authors of Potes et al. (24). Although Mel-frequency cepstrum coefficients (25) (MFCCs) were developed to mimic human perception and are widely used in speech recognition, they have been shown to work for heart sound analysis as well (24, 26–28). We extracted the first 13 coefficients from each of the four states. The “*2–5”* bands of the spectral contrast features include: 25–50, 50–100, 100–200, and 200–400 Hz. Daubechies 4 wavelet was used as a basis for the discrete wavelet transform features.

To smooth out the outliers, we generated another set of 2 × 177 features representing the mean and standard deviation of the features taken as a sliding window with window size six across the segments of each PCG. To ensure that each segment was equally represented, windowing was performed cyclically. The resulting 354 features were then used for the models’ evaluation.

The features were extracted and calculated using Python 3.7 and libraries Librosa 0.9.1 (29), Scipy 1.5.2 (SciPy, RRID:SCR_008058) (30) and Numpy 1.18.5 (NumPy, RRID:SCR_008633) (31).

#### Experimental pipeline

We implemented 10 ML models. The decision tree classifier (DT) is a model that uses a tree diagram for decision making, where each branch is partitioned based on a threshold for a feature. The gradient boosting classifier (GB), the extreme gradient boosting classifier (XGB), the light gradient boosting machine classifier (LGBM), and the random forest classifier (RF) are ensemble methods that combine the predictions of multiple DTs. The C-support vector classifier (SVC) finds a hyper-plane in the feature space that spatially separates the classes. The K-neighbors classifier (KN) looks for closest neighbors in the features space to determine the class. The Gaussian naive Bayes (GNB) utilizes Bayes’ theorem and makes the assumption that the features are independent and described by a Gaussian distribution. The logistic regression (LR) uses logistic function to map a linear combination of the features to a value between 0 and 1. The stochastic gradient descent classifier (SGD) takes iterative steps to minimize the cost function. All of the implemented models are probabilistic, meaning they assign probabilities for each class. The selected decision threshold for all models was 0.5. Each PCGs final decision was selected as the majority vote of the segments’ predictions. The models were implemented using Python 3.7 and Scikit 0.24.2 (scikit-learn, RRID:SCR_002577) (32) and lightgbm 3.3.1 (LightGBM, RRID:SCR_021697) (33) libraries.

Models were evaluated with a subject-wise 10-fold cross validation using stratified folds with respect to the two different setups for data acquisition. We found that the models, trained only on the PCGs that were correctly segmented, perform significantly better. Thus, for each training set, we removed subjects that correspond to one of the seven PCGs we manually determined are segmented incorrectly.

To keep the models explainable and as transparent as possible and to avoid overfitting, we performed feature selection, retaining only a subset of the features used as model input. Although the selected features can depend on the model (e.g., decision tree based models can calculate the importance of the features according to their ability to increase the pureness of the levels), we selected our features independently of the models. This means that all of the models used the same selected features. Features were selected by calculating the mutual information (34) between each feature and the outcome variable. The mutual information between two variables is zero if the two variables are independent, and higher values indicate greater dependence. Each training fold was divided into five stratified subfolds, and 40 features that had the highest mutual information with the outcome variable on average across the five subfolds were used for training.

## Results

Patient clinical characteristics are outlined in Table 3. Our final dataset included 898 decompensated and 908 recompensated (1,806 in total) PCG segments with 354 features. We used accuracy, precision, recall, F1-score, and area under receiver operating curve (ROC AUC) as the evaluation metrics, with accuracy as the main metric of performance evaluation. Formulas for calculation of accuracy, precision, recall, and F1-score are given in Equations (1–4). TP, FP, TN, and FN denote true positive, false positive, true negative, and false negative, respectively.

**TABLE 3:** Patient clinical characteristics.

Parameter	Study population (*N* = 37)
Age, y	56.6 ± 12
Gender (male), %	88
Heart failure etiology (ischemic), %	27
**Cause of decompensation**	
Volume overload, %	78
Infection, %	15
Arrhythmia, %	7
LVEF, %	26.7 ± 7.7
NT-proBNP, pg/ml	4,593 (953, 5,102)
**Medical therapy**	
ARNI/ACEI/ARB, %	96
Beta blockers, %	100
MRA, %	96
SGLT2i, %	70
Diuretic, %	96
Ca-antagonist, %	9
Digoxin, %	15

LVEF, left ventricular ejection fraction; ARNI, angiotensin receptor antagonist neprylisin inhibitor; ACEI, angiotensin convertase enzyme inhibitor; ARB, angiotensin receptor blocker; MRA, mineralocorticoid receptor blocker; SGLT2i, sodium glucose transporter 2 inhibitor; Ca, calcium.


(1) $$accuracy=\frac{TP+TN}{TP+TN+FP+FN}$$

(2) $$precision=\frac{TP}{TP+FP}$$

(3) $$recall=\frac{TP}{TP+TN}$$

(4) $$F1=2\cdot \frac{precision\cdot recall}{precision+recall}$$

### Classification by the experts

The baseline of our method was determined by three cardiologists experts who were each asked to independently listen to a representative subset of 12 PCG recordings and classify them as decompensated or recompensated. Importantly, no other clinical data on the CHF patients were available to the clinicians at that time. This subset included three decompensated and three recompensated recordings from each of the two data acquisition setups. The results are given in Table 4.

**TABLE 4:** Results of classification of a representative subset of our dataset by the medical experts.

PCG	Class	Expert 1	Expert 2	Expert 3	Accuracy
1	1	0	1	0	0.33
2	0	1	1	1	0
3	1	1	0	0	0.33
4	1	1	1	1	1
5	0	0	0	0	1
6	0	0	0	1	0.67
7	0	1	1	1	0
8	1	1	0	0	0.33
9	1	0	1	0	0.33
10	0	0	0	1	0.67
11	0	0	0	0	1
12	1	0	1	0	0.33
Overall accuracy		0.58	0.67	0.25	0.5

Classes 0 and 1 are recompensated and decompensated CHF phases, respectively.

The experts’ classification accuracies were 58, 67, and 25%, averaging at 50%, which coincides with the dataset class distribution, meaning the cardiac auscultation alone contributes little to the experts’ recognition of CHF decompensation episode.

### Evaluation the models’ performance

The results of the models’ performance evaluation are shown in Table 5. The results are given along with standard deviation (SD) and t-distribution 95% confidence interval (CI).

**TABLE 5:** Results of the models’ performance.

Classifier	Accuracy	Precision	Recall	F1	ROC AUC
LR	**0.72 (0.15; 0.61–0.83)**	0.73 (0.17; 0.61–0.86)	**0.73 (0.22; 0.56–0.90)**	**0.71 (0.16; 0.59–0.84)**	**0.74 (0.18; 0.61–0.88)**
LGBM	0.70 (0.16; 0.58–0.82)	0.68 (0.29; 0.47–0.90)	0.63 (0.29; 0.41–0.85)	0.63 (0.27; 0.43–0.84)	0.71 (0.16; 0.59–0.83)
SVC	0.68 (0.16; 0.56–0.79)	**0.75 (0.23; 0.58–0.92)**	0.60 (0.31; 0.37–0.84)	0.62 (0.22; 0.45–0.78)	0.71 (0.15; 0.59–0.83)
RF	0.68 (0.18; 0.54–0.81)	0.63 (0.28; 0.42–0.84)	0.68 (0.35; 0.42–0.94)	0.63 (0.28; 0.41–0.84)	0.66 (0.23; 0.48–0.83)
DT	0.62 (0.15; 0.51–0.73)	0.60 (0.25; 0.41–0.79)	0.66 (0.29; 0.44–0.87)	0.60 (0.23; 0.42–0.78)	0.66 (0.16; 0.53–0.78)
GB	0.61 (0.16; 0.48–0.73)	0.61 (0.25; 0.42–0.80)	0.63 (0.27; 0.43–0.83)	0.59 (0.23; 0.42–0.77)	0.65 (0.17; 0.52–0.77)
XGB	0.61 (0.19; 0.46–0.75)	0.59 (0.27; 0.39–0.80)	0.60 (0.31; 0.37–0.84)	0.58 (0.26; 0.38–0.77)	0.67 (0.18; 0.53–0.81)
KN	0.58 (0.16; 0.47–0.70)	0.60 (0.19; 0.45–0.75)	0.58 (0.23; 0.40–0.75)	0.57 (0.18; 0.43–0.71)	0.61 (0.17; 0.48–0.74)
SGD	0.58 (0.07; 0.52–0.63)	0.45 (0.23; 0.28–0.62)	0.71 (0.41; 0.40–1.02)	0.54 (0.29; 0.32–0.76)	0.63 (0.13; 0.53–0.73)
GNB	0.54 (0.14; 0.44–0.65)	0.52 (0.21; 0.37–0.68)	0.55 (0.27; 0.35–0.75)	0.52 (0.21; 0.36–0.68)	0.57 (0.23; 0.39–0.74)

The scores are given as mean (SD; 95% CI).

The results are calculated from 10-fold cross-validation.

The highest version of the individual metric is marked as bold.

**FIGURE 2:**
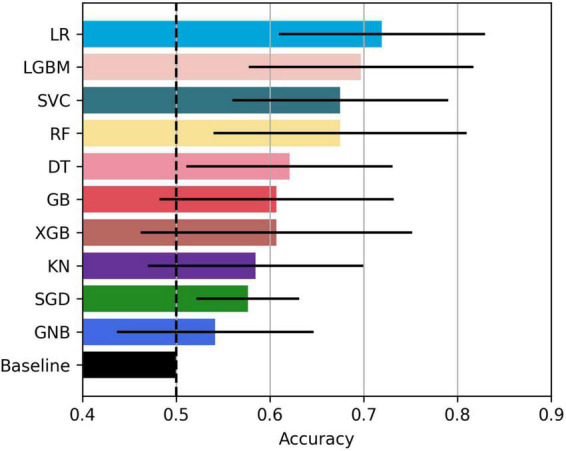
Bar plot of the model accuracies. Black horizontal lines represent 95% CI.

**FIGURE 3:**
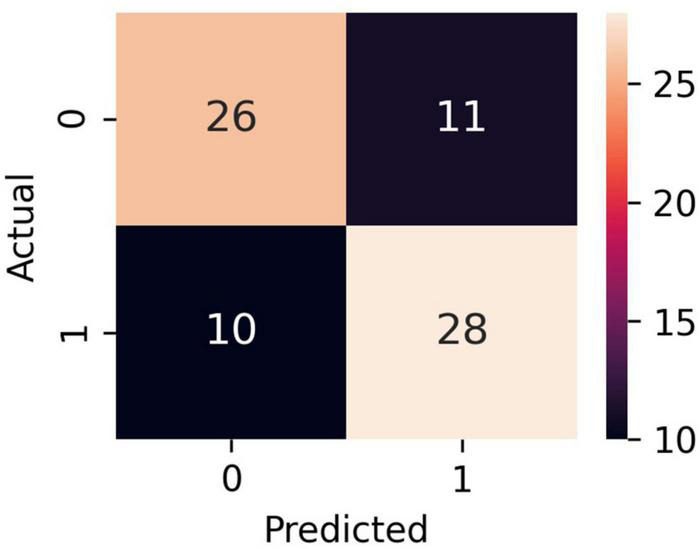
Confusion matrix of the LR model. Classes 0 and 1 are recompensated and decompensated CHF phases, respectively.

**FIGURE 4:**
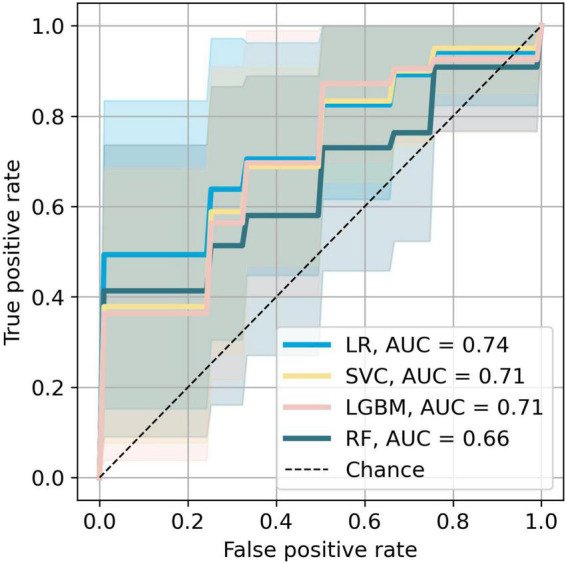
Receiver operating characteristic curves of the most accurate four models. The colored areas represent the 95% CI.

The bar plot of the models’ accuracies is shown in Figure 2. All of the 10 implemented models outperform the baseline, while six of them outperform the baseline with the 95% CI. The best performing model is LR, which achieved accuracy (SD; 95% CI) of 0.72 (0.15; 0.61–0.83). Additionally, the LR model also achieved highest performance in recall with the score of 0.73 (0.22; 0.56–0.90), F1-score of 0.71 (0.16; 0.59–0.84), and ROC AUC with the score of 0.74 (0.18; 0.61–0.88). The confusion matrix of the LR is shown in Figure 3. The best performing model according to the precision metric was SVC, which achieved a score of 0.75 (0.28; 0.58–0.92). The models with accuracies comparable to that of the LR model are LGBM, which achieved the score of 0.70 (0.16; 0.59–0.82), SVC, which achieved the score of 0.68 (0.16; 0.56–0.79), and RF, which achieved the score of 0.68 (0.18; 0.51–0.73). The ROC curves of the four most accurate models are shown in Figure 4.

To test whether the difference in the models’ predictive accuracy was statistically significant, we calculated the *p*-values from McNemar’s test (35). This test is used on contingency tables of the two models’ predictions. The results are given in Table 6. We see that all of the models provided similar predictions, since the *p*-values are all close to 1.

**TABLE 6:** *P*-values of McNemar’s tests between the ML models.

	LR	LGBM	SVC	RF	DT	GB	XGB	KN	SGD	GNB
LR	/	0.91	0.88	0.81	0.79	0.74	0.76	0.72	0.83	0.78
LGBM	0.91	/	0.85	0.9	0.91	0.9	0.9	0.78	0.84	0.75
SVC	0.88	0.85	/	0.88	0.86	0.88	0.88	0.86	0.82	0.78
RF	0.81	0.9	0.88	/	1	0.85	0.9	0.86	0.85	0.86
DT	0.79	0.91	0.86	1	/	1	1	0.95	0.92	0.91
GB	0.74	0.90	0.88	0.85	1	/	0.95	0.90	0.86	0.85
XGB	0.76	0.9	0.88	0.9	1	0.95	/	0.85	0.85	0.85
KN	0.72	0.78	0.86	0.86	0.95	0.9	0.85	/	0.85	0.89
SGD	0.83	0.84	0.82	0.85	0.92	0.86	0.85	0.85	/	0.91
GNB	0.78	0.75	0.78	0.86	0.91	0.85	0.85	0.89	0.91	/

The list of the top 40 features is found in Table 7, with the most important features being time domain features (10/40), power spectral density features for different frequency bands (17/40), and MFCCs (9/40). The most important heart sound seems to be diastole, as 21/40 of the most important features were extracted from diastole.

**TABLE 7:** Top 40 best predictor features according to their mutual information with the outcome.

Rank	Feature	MI	Rank	Feature	MI
1	m_BPM	0.16	21	m_Dur_Ratio_S1RR	0.1
2	m_Dur_Ratio_DiaRR	0.15	22	m_PSD_Dia_140_160Hz	0.1
3	m_Dur_Dia	0.14	23	sd_PSD_Sys_250_300Hz	0.1
4	sd_PSD_Dia_200_250Hz	0.14	24	sd_PSD_Sys_200_250Hz	0.1
5	sd_BPM	0.14	25	m_mfcc6_Dia	0.1
6	m_Dur_Ratio_SysS1	0.13	26	m_ZC_Dia	0.1
7	m_mfcc4_Dia	0.13	27	sd_PSD_Sys_140_160Hz	0.1
8	m_Dur_Ratio_SysDia	0.13	28	m_PSD_Dia_300_400Hz	0.1
9	sd_PSD_Dia_250_300Hz	0.13	29	m_PSD_Sys_300_400Hz	0.1
10	m_mfcc2_Sys	0.12	30	m_SpecContrast5_Dia	0.09
11	sd_PSD_Dia_180_200Hz	0.12	31	m_mfcc1_Sys	0.09
12	sd_PSD_Dia_160_180Hz	0.12	32	m_SpecCentroid_Dia	0.09
13	sd_PSD_Sys_120_140Hz	0.12	33	m_SpecBandwidth_Dia	0.09
14	m_mfcc2_Dia	0.12	34	m_mfcc6_S1	0.09
15	sd_Dur_Ratio_SysS1	0.11	35	sd_PSD_Sys_300_400Hz	0.09
16	m_mfcc1_Dia	0.11	36	m_PSD_Dia_250_300Hz	0.09
17	sd_PSD_Dia_140_160Hz	0.11	37	m_mfcc4_Sys	0.09
18	sd_PSD_Sys_180_200Hz	0.11	38	m_Dur_Ratio_SysRR	0.09
19	m_PSD_Dia_160_180Hz	0.11	39	m_Dur_Sys	0.09
20	m_mfcc6_Sys	0.11	40	sd_PSD_Dia_100_120Hz	0.08

The prefixes “m_” and “sd_” correspond to the mean and standard deviation of the features taken as a sliding window with window size six across the segments of each PCG. BPM, beats per minute; Dur, duration; PSD, power spectral density; mfcc, Mel-frequency cepstral coefficients; ZC, zero crossings; SpecContrast, spectral contrast; SpecCentroid, spectral centroid; SpecBandwidth, spectral bandwidth.

### PhysioNet dataset experiments

To test the robustness of our pipeline, we tested it against dataset A of the PhysioNet (PhysioNet, RRID:SCR_007345) (36) public database of heart sound recordings. The dataset A contains PCGs from 117 normal and 292 abnormal hearts recorded from children and adults. Both healthy subjects and patients contributed between one and six PCGs. The recordings lasted between 9 and 36 s. The models were compared in a 10-fold cross validation with folds stratified with respect to the class. The model with the best accuracy was SVC, which achieved a score of 0.80 (0.06; 0.76–0.85), better than that of the majority, which was 0.71. The other best performing models were LGBM, GB, and XGB, with the accuracy scores of 0.80 (0.07; 0.74–0.85), 0.78 (0.08; 0.72–0.84), and 0.78 (0.06; 0.73–0.83), respectively. The results obtained with this method are very similar to those obtained with our previous approach in (11). It should be noted that the results may be somewhat positively biased as the subjects are not labeled and the recordings of the same subjects may be included in both the training and test sets.

## Discussion

In this study, we used 10 ML models to classify decompensation episodes in CHF using a dataset of heart sound recordings from 37 CHF patients. We used 40 domain predictor features, extracted from the four states of heart sounds. All models outperformed the classification performed independently by three cardiology experts, which averaged at 50%. Logistic regression proved to be the best model in terms of accuracy, reaching 72 (15; 61–83)%. Power spectral density features, time domain features, and Mel-frequency cepstrum coefficients were found to be the most important predictors. Most of these features were extracted from diastole. From the medical perspective, this is reasonable, since the sounds produced in the diastole originate from the heart chambers refilling by blood. The heart of a CHF patient is more rigid than a healthy heart and will thus vibrate differently. Another observation is that several of the important features are related to heart rate (BMP). Again, this is relevant from the medical point of view, as patients in the decompensated phase have a faster pulse than those that are not decompensated. Our method was additionally tested on a public dataset of normal/abnormal heart sounds where it achieved exemplary results, although a direct comparison is not possible because the public dataset was heavily unbalanced.

In view of early detection of decompensation episodes of CHF and thus preventing decompensation from occurring or to occur in milder forms that would not lead to hospitalization, the results are promising, as they demonstrate that the ML algorithms can substantially outperform a human expert solely based on the heart sounds. It is important to stress that the algorithms were trained on data from the two extreme phases of CHF, indicating that the results obtained likely represent the upper limit on the accuracy such an approach can achieve. Thus, using only heart sounds for detection of decompensation is not sufficient, however, it can represent a valuable component of a decision-support system that takes into account additional patient data, with patients performing daily/weekly self-recording and self-assessment.

Recently, various approaches to automatic detection of heart disease have been successfully implemented for numerous data set modalities such as clinical features, images, and electrocardiograms (ECG) (37). Although the reported accuracies are very decent, some data are very difficult and/or expensive to obtain. Future plan for our system to support patients with CHF is to incorporate data that are easy to obtain and relatively inexpensive, such as clinical data, self-reported data, ECG data, daily activity data, and possibly others. In addition, our models could be integrated into a virtual coaching system (38) that tracks the patient’s cardiac status and overall well-being and promotes medication use and/or physical activity to prevent deterioration of the condition.

### Limitations

This study has the following limitations. First, the recorded patients are at different stages of CHF so a decompensated phase of a relatively healthy CHF patient can be similar to a recompensated phase of a patient with a later stage of CHF. In addition, there are different subtypes of CHF, which we did not consider in model building. Models trained separately for each stage/subtype would most likely provide better results. Second, the PCG is recorded when an individual is admitted to/discharged from the hospital, and not on a regular basis with the intention of capturing the deterioration of the condition. Deterioration is unpredictable, and therefore data collection starting with a CHF patient in good condition and then waiting until the situation deteriorates is not practical. Third, since we are using a dataset collected by ourselves, we cannot directly compare the accuracy of our method with related work, but only by testing it against a public dataset. Fourth, although ML models outperform the experts’ classification, the inputs to the models are computer-extracted sound features most of which are not intuitive to the experts. Therefore, the models do not really provide the experts with additional knowledge to help them make decisions while listening to the heart, but can only be used as a component of stand-alone decision-making tools.

## Conclusion

This study demonstrates that in chronic heart failure patients machine learning algorithms may outperformcardiologists in detecting decompensation episodes based on heart sounds alone. The key predictor features are derived from diastole and come both from time and frequency domains. Although the results are promising, showing that machine learning algorithms perform better than cardiology experts, the use of heart sound data alone is not sufficient for early detection of decompensation. Therefore, additional clinical data must be added to the protocol before considering the integration of this method into a decision-support system. The inclusion of additional predictor variables such as weight, self-reported data, and electrocardiogram falls within the scope of future work.

## Data availability statement

Raw data will be made available upon request.

## Ethics statement

The studies involving human participants were reviewed and approved by Republic of Slovenia National Medical Ethics Committee. Written informed consent for participation was not required for this study in accordance with the national legislation and the institutional requirements.

## Author contributions

GP and AG designed the study. GP collected the data. DS developed the algorithms and performed the analysis. All authors participated in the analysis of the results and wrote the manuscript.

## References

[1] McDonagh TA Metra M Adamo M Gardner RS Baumbach A Böhm M 2021 ESC Guidelines for the diagnosis and treatment of acute and chronic heart failure. *Eur Heart J.* (2021) 42:3599–726. 10.1093/eurheartj/ehab368 34447992

[2] ClinicalTrials.gov. *CardioMEMS Heart Sensor Allows Monitoring of Pressure to Improve Outcomes in NYHA Class III Heart Failure Patients. ClinicalTrials.gov identifier: NCT00531661.* (2015). Available online at: https://clinicaltrials.gov/ct2/show/study/NCT0053166 (accessed July 25, 2022).

[3] Jahmunah V Oh SL Wei JKE Ciaccio EJ Chua K San TR Computer-aided diagnosis of congestive heart failure using ECG signals – A review. *Phys Med.* (2019) 62:95–104. 10.1016/j.ejmp.2019.05.004 31153403

[4] Bhurane AA Sharma M San-Tan R Acharya UR. An efficient detection of congestive heart failure using frequency localized filter banks for the diagnosis with ECG signals. *Cogn Syst Res.* (2019) 55:82–94. 10.1016/j.cogsys.2018.12.017

[5] Tripoliti EE Papadopoulos TG Karanasiou GS Naka KK Fotiadis DI. Heart failure: diagnosis, severity estimation and prediction of adverse events through machine learning techniques. *Comput Struct Biotechnol J.* (2017) 15:26–47. 10.1016/j.csbj.2016.11.001 27942354 PMC5133661

[6] Liu C Springer D Li Q Moody B Juan RA Chorro FJ An open access database for the evaluation of heart sound algorithms. *Physiol Meas.* (2016) 37:2181–213. 10.1088/0967-3334/37/12/2181 27869105 PMC7199391

[7] Clifford GD Liu C Moody B Millet J Schmidt S Li Q Recent advances in heart sound analysis. *Physiol Meas.* (2017) 38:10–25. 10.1088/1361-6579/aa7ec8 28696334 PMC11460977

[8] Sidra G Ammara N Taimur H Bilal H Ramsha A. Fully automated identification of heart sounds for the analysis of cardiovascular pathology. In: Khan F Jan MA Alam M editors. *Applications of Intelligent Technologies in Healthcare.* Cham: Springer (2018). p. 117–29.

[9] Dwivedi AK Imtiaz SA Rodriguez-Villegas E. Algorithms for automatic analysis and classification of heart sounds–a systematic review. *IEEE Access.* (2019) 7:8316–45. 10.1109/ACCESS.2018.2889437

[10] Gjoreski M Simjanoska M Gradišek A Peterlin A Gams M Poglajen G. Chronic heart failure detection from heart sounds using a stack of machine-learning classifiers. In: Jason J editor. *Proceedings of the 13th International Conference on Intelligent Environments; 2017 Aug 23-25.* Seoul: IEEE (2017). p. 14–9.

[11] Gjoreski M Gradišek A Budna B Gams M Poglajen G. Machine learning and end-to-end deep learning for the detection of chronic heart failure from heart sounds. *IEEE Access.* (2020) 8:20313–24. 10.1109/ACCESS.2020.2968900

[12] Gao S Zheng Y Guo X. Gated recurrent unit-based heart sound analysis for heart failure screening. *Biomed Eng.* (2020) 19:3. 10.1186/s12938-020-0747-x 31931811 PMC6958660

[13] Liu Y Guo X Zheng Y. An automatic approach using ELM classifier for HFpEF identification based on heart sound characteristics. *J Med Syst.* (2019) 43:285. 10.1007/s10916-019-1415-1 31309299

[14] Zheng Y Guo X Qin J Xiao S. Computer-assisted diagnosis for chronic heart failure by the analysis of their cardiac reserve and heart sound characteristics. *Comput Methods Programs Biomed.* (2015) 112:372–83. 10.1016/j.cmpb.2015.09.001 26387633

[15] Susic̆ D Poglajen G Gradišek A. Machine learning models for detection of decompensation in chronic heart failure using heart sounds. In: Humberto H Valera A Luštrek M editors. *Proceedings of the Workshops at 18th International Conference on Intelligent Environments (IE2022).* Amsterdam: IOS Press (2022). p. 340–9.

[16] Gjoreski M Gradišek A Budna B Gams M Poglajen G. Toward early detection and monitoring of chronic heart failure using heart sounds. In: Muñoz A Ouhbi S Minker W Echabbi L Navarro-Cía M editors. *Proceedings of the 15th International Conference on Intelligent Environments in conjunction with the 15th International Conference on Intelligent Environments (IE19); 2019 Jun 24-27; Rabat, Morocco (Ambient intelligence and smart environments, 26).* Amsterdam: IOS Press (2019). p. 336–43.

[17] Littmann Electronic Stethoscopes 3M United States. *Littmann Electronic Stethoscopes.* (2022). Available online at: https://www.littmann.com/3M/en_US/littmann-stethoscopes/products/~/3M-Littmann-Stethoscopes/Electronic-Stethoscopes/?N=5142935+8711017+8727094+3294857497&rt=r3 (accessed July 13 2022).

[18] Eko. *Eko DUO ECG + Digital Stethoscope.* (2022). Available online at: https://shop.ekohealth.com/products/duo-ecg-digital-stethoscope?variant=39350415655008 (accessed July 13, 2022).

[19] McGee S. Auscultation of the heart: general principles. 5th ed. *Evidence-Based Physical Diagnosis.* Philadephia, PA: Elsevier (2022). p. 327–32.

[20] Springer DB Tarassenko L Clifford GD. Logistic regression-HSMM-based heart sound segmentation. *IEEE Trans Biomed Eng.* (2016) 63:822–32. 10.1109/TBME.2015.2475278 26340769

[21] Schmidt SE Holst-Hansen C Graff C Toft E Struijk JJ. Segmentation of heart sound recordings by a duration-dependent hidden Markov model. *Physiol Meas.* (2010) 31:513–29. 10.1109/CIC.2008.4749049 20208091

[22] Robert J Webbie M. *Pydub [Internet].* San Francisco, CA: GitHub (2018)

[23] Matlab. *version 9.10.0 (R2021a).* Natick, MA: The MathWorks Inc (2021).

[24] Potes C Parvaneh S Rahman A Conroy B. Ensemble of feature-based and deep learning-based classifiers for detection of abnormal heart sounds. *Proceedings of the 2016 Computing in Cardiology Conference (CinC).* Vancouver, BC: IEEE (2016). p. 621–4. 10.22489/CinC.2016.182-399

[25] Rabiner L Juang BH. *Fundamentals of Speech Recognition.* Englewood Cliffs, NJ: Prentice-Hall (1993).

[26] Zabihi M Rad AB Kiranyaz S Gabbouj M Katsaggelos AK. Heart sound anomaly and quality detection using ensemble of neural networks without segmentation. *Proceedings of the 2016 Computing in Cardiology Conference (CinC).* Vancouver, BC: IEEE (2016). p. 613–6. 10.22489/CinC.2016.180-213

[27] Chen TE Yang SI Ho LT Tsai KH Chen YH Chang YF S1 and S2 heart sound recognition using deep neural networks. *IEEE Trans Biomed Eng.* (2017) 64:372–80. 10.1109/TBME.2016.2559800 28113191

[28] Nilanon T Yao J Hao J Purushotham S Liu Y. Normal / abnormal heart sound recordings classification using convolutional neural network. *Proceedings of the 2016 Computing in Cardiology Conference (CinC).* Vancouver, BC: IEEE (2016). p. 585–8. 10.22489/CinC.2016.169-535

[29] McFee B Metsai A McVicar M Balke S Thomé C Raffel C *Librosa/Librosa: 0.9.1 [Internet].* (2022). Available online at: https://zenodo.org/record/6097378 (accessed February 15, 2022).

[30] Virtanen P Gommers R Oliphant TE Haberland M Reddy T Cournapeau D SciPy 1.0: fundamental algorithms for scientific computing in Python. *Nat Methods.* (2020) 17:261–72. 10.1038/s41592-019-0686-2 32015543 PMC7056644

[31] Harris CR Millman KJ van der Walt SJ Gommers R Virtanen P Cournapeau D Array programming with NumPy. *Nature.* (2020) 585:357–62. 10.1038/s41586-020-2649-2 32939066 PMC7759461

[32] Pedregosa F Varoquaux G Gramfort A Michel V Thirion B Grisel O Scikit-learn: machine learning in Python. *J Mach Learn Res.* (2011) 12:2825–30.

[33] Ke G Meng Q Finley T LightGBM: a highly efficient gradient boosting decision tree. In: Jordan MI LeCun Y Solla SA editors. *Advances in Neural Information Processing Systems.* Red Hook, NY: Curran Associates, Inc (2017). 30 p. 10.1016/j.envres.2020.110363

[34] Kraskov A Stögbauer H Grassberger P. Estimating mutual information. *Phys Rev E.* (2004) 69:066138. 10.1103/PhysRevE.69.066138 15244698

[35] McNemar Q. Note on the sampling error of the difference between correlated proportions or percentages. *Psychometrika.* (1947) 12:153–7. 10.1007/bf02295996 20254758

[36] Goldberger AL Amaral LA Glass L Hausdorff JM Ivanov PC Mark RG PhysioBank, PhysioToolkit, and PhysioNet: components of a new research resource for complex physiologic signals. *Circ.* (2000) 101:215–20. 10.1161/01.cir.101.23.e215 10851218

[37] Javeed A Khan SU Ali L Ali S Imrana Y Rahman A. Machine learning-based automated diagnostic systems developed for heart failure prediction using different types of data modalities: a systematic review and future directions. *Comput Math Methods Med.* (2022) 2022:1–30. 10.1155/2022/9288452 35154361 PMC8831075

[38] Tsiouris KM Tsakanikas VD Gatsios D Fotiadis DI. A review of virtual coaching systems in healthcare: closing the loop with real-time feedback. *Front Digit Health.* (2020) 2:567502. 10.3389/fdgth.2020.567502 34713040 PMC8522109

